# Family Stressors and Resources as Social Determinants of Health among Caregivers and Young Children

**DOI:** 10.3390/children9040452

**Published:** 2022-03-23

**Authors:** Natalie Slopen, Benjamin Le Cook, Justin Winston Morgan, Michael William Flores, Camila Mateo, Cynthia Garcia Coll, Dolores Acevedo Garcia, Naomi Priest, Elaine Wethington, Esther Lee, Margo Moyer, Nathaniel M. Tran, Sandra Krumholz, David R. Williams

**Affiliations:** 1 Department of Social and Behavioral Sciences, Harvard T. H. Chan School of Public Health, Boston, MA 02115, USA; jmorgan@g.harvard.edu (J.W.M.); krumholz@hsph.harvard.edu (S.K.); dwilliam@hsph.harvard.edu (D.R.W.); 2 Center on the Developing Child, Harvard University, Cambridge, MA 02138, USA; 3 Health Equity Research Lab, Cambridge Health Alliance, Cambridge, MA 02139, USA; bcook@challiance.org (B.L.C.); mwflores@challiance.org (M.W.F.); moyerma1@gmail.com (M.M.); 4 Harvard Medical School, Boston, MA 02115, USA; camila.mateo@childrens.harvard.edu; 5 Department of Pediatrics, University of Puerto Rico, San Juan 00935, Puerto Rico; cynthia.garciacoll@upr.edu; 6 Institute for Child, Youth and Family Policy, Heller School for Social Policy and Management, Brandeis University, Waltham, MA 02454, USA; dacevedo@brandeis.edu; 7 Center for Social Research and Methods, Australia National University, Canberra 0200, Australia; naomi.priest@anu.edu.au; 8 Population Health, Murdoch Children’s Research Centre, Royal Children’s Hospital, Melbourne 3025, Australia; 9 Survey Research Center, Institute for Social Research, University of Michigan, Ann Arbor, MA 48106, USA; ew20@cornell.edu; 10 University of Michigan School of Public Health, Ann Arbor, MI 48109, USA; esylee@umich.edu; 11 Department of Health Policy, Vanderbilt University, Nashville, TN 37203, USA; nathaniel.tran@vanderbilt.edu; 12 Departments of African and African American Studies and of Sociology, Harvard University, Cambridge, MA 02138, USA

**Keywords:** stressors, protective factors, children, caregivers, health

## Abstract

Life course-informed theories of development suggest it is important to integrate information about positive and negative aspects of the social environment into studies of child and parental wellbeing, including both stressors that compromise health and resources that promote well-being. We recruited a sample of 169 pairs of caregivers and young children (birth to 5 years) from a community health clinic and administered survey questions to assess stressors and resources. We constructed inventories of stressors and resources and examined the relationships between these inventories and caregivers’ depressive symptoms, anxiety symptoms, and sleep problems, and young children’s medical diagnoses derived from electronic health records. Cumulative stressors and resources displayed bivariate and adjusted associations with caregivers’ depressive symptoms, anxiety symptoms, and sleep problems. For depressive and anxiety symptoms, these associations were evident in models that included stressors and resources together. Caregivers with high stressors and low resources displayed the highest levels of depressive and anxiety symptoms and sleep problems. In terms of children’s health outcomes, only modest trends were evident for developmental/mental health outcomes, but not other diagnostic categories. Future studies are needed to examine stressors and resources together in larger samples and in relation to prospectively assessed measures of child well-being.

## 1. Introduction

Social context affects the health and wellbeing of parents and children, including stressors that compromise health and resources that promote well-being [1]. The interplay of compounding stressors and resources, shaped by the social determinants of health, influences healthy development and also health disparities by race/ethnicity and socioeconomic status, which emerge early in life and widen over time [2]. The accurate measurement of stressors and resources that affect infants and young children requires a comprehensive assessment of experiences in their parents’ lives and an understanding of the broader social context in which families reside [3,4]. Within life course theory, an accumulation model suggests that the occurrence of multiple risk exposures exceeds the negative developmental consequences of a single exposure [5], often displaying a graded positive relationship [6,7,8,9]. More recently, studies have shown that an accumulation model also applies to the accrual of resources or assets in the family, with additional protective factors associated with a lower risk of poor health or increased well-being and resilience [10,11]. Whereas most research using an additive model has focused on either the accumulation of stressors [6] or resources [12], a variety of theories and frameworks within developmental psychology emphasize the importance of studying risks and protective factors together [13].

Several recent studies have examined childhood stressors and resources together for health outcomes in children [14,15] and adults [11,16,17]. For example, in a nationally representative study with children aged 8 to 17 (*n* = 40,302), adverse childhood experiences and positive childhood experiences interacted, whereby children who were exposed to high levels of adversity and low positive experiences had over 8 times the odds of depression compared to children with low adversity and high positive experiences [14]. In another study (*n* = 489, ages 10–13 years at baseline), researchers found that the ratio of childhood adversities to protective factors was associated with a range of health outcomes nearly ten years later [16]. Similar patterns are evident when outcomes are assessed in adulthood: for example, using data from a random digit-dial telephone survey of over 6000 adults in Wisconsin, positive childhood experiences were associated with a lower risk of depression, adjusting for adverse childhood experiences [11]. These studies align with other studies illustrating that positive social experiences can protect against the adverse health consequences of stressors [17,18,19,20]. In the present study, we build on existing research to examine stressors and resources together in relation to outcomes in young children and the parents of young children.

Social scientists and health practitioners have developed a wide array of options for high-quality measures of distinct family processes, attributes, and events relevant for characterizing the lives of caregivers and young children. Validated measures for specific parent or family characteristics, experiences, or contexts are typically long (e.g., ≥10 items), making it challenging to bring together multiple measures to characterize caregiver and young children’s social environments comprehensively within research or clinical contexts, and to incorporate multiple detailed, multi-item assessments into analyses. With the increasing interest in integrating information about positive and negative aspects of children’s social environment into research studies and clinical practices [11,21,22,23,24,25], there is a need for brief yet robust measures to capture this information.

Guided by an accumulation model [6], in the present study, we constructed inventories of cumulative risk and protective factors using short scales and single-item questions to reflect stressors and protective factors across multiple domains relevant to children’s development from infancy through five years of age and their parent’s wellbeing. We focused this study on young children and their parents, given the importance of these earliest years for healthy development over time.

## 2. Methods

### 2.1. Participants

Our study team recruited 169 caregiver-child dyads from the waiting room of a single community health center in Massachusetts. Families were eligible for inclusion in this cross-sectional study if the adult was a primary caregiver to a young child (ages 0 to 5 years) present in the clinic for an appointment. If eligible, the primary caregiver completed an informed consent process and the survey in English, Spanish, Portuguese, or Haitian Creole (i.e., the four most common languages spoken in this clinic population). Caregivers completed the surveys via a tablet while at the clinic, with help from research assistants as needed. Following survey completion, the research assistant added the child’s medical record number to the survey record to make it possible to link the child’s electronic health record (EHR) to the survey responses. The Institutional Review Board at Cambridge Health Alliance approved the study.

### 2.2. Measures

We collected information on a broad range of stressors and resources that can impact opportunities for healthy child development until five years of age. Experts from psychology, sociology, public health, and human development (some included in this authorship group) were asked to recommend brief yet validated questions for a range of stressors and resources recognized as central to parental wellbeing and child health. The overall goal was to create a survey with high-quality brief measures. The included constructs and associated measures were selected based on the current theories of early child development and research building on these models [1,26,27,28], structured discussions, and eventual consensus among these experts. We retained the original items and response values, which are not consistent across items; as described below, following other research that has combined information across multiple measures with different scales [29,30], we created dichotomous variables for each construct to represent high or low values for each stressor or resource (described in more detail below). This approach to developing cumulative scores is widespread within child development research, as it is (a) parsimonious, (b) statistically sensitive with small samples, (c) avoids assumptions about the relative strengths of the component factors or their collinearity, and (d) fits with underlying theoretical models that suggest that multiple exposures are more potent than single exposures [6].

#### 2.2.1. Cumulative Stressor Inventory

We constructed a cumulative stressor inventory that included 16 dichotomous indicators (range 0–16), created from the items listed in Table 1. The 16 included indicators reflect: (1) perceived stress in the past 12 months (1 Likert-style item); (2) major life events (5 dichotomous items) [31]; (3) everyday discrimination (5 Likert-style items) [32]; (4) housing instability (1 count item); (5) job instability (1 count item); (6) work schedule instability (1 Likert-style item); (7) job insecurity (1 Likert-style item); (8) work–life balance stress (1 Likert-style item); (9) financial insecurity (1 Likert-style item); (10) negative religious coping (2 Likert-style items); (11) neighborhood safety (1 Likert-style item); (12) family immigration concerns (1 Likert-style items, adapted from the National Survey of Hispanics); 26 stressful events during (13) the 12 months prior to conception of the child attending the clinic, (14) during pregnancy, and (15) since the child was born (with events selected from a variety of sources); and, (16) adverse childhood experiences of the respondent (8 dichotomous items) [33]. Table A1 in Appendix A presents correlations between the stressors, means, standard deviations, and ranges. In addition, this table shows the thresholds for dichotomization of each of the 16 indicators for use in the cumulative stressor score (i.e., top quartile, or closest approximation), as well as Cronbach’s alpha for the Likert-style measures with multiple items. To create the cumulative stressor inventory score, we summed the indicator variables created for each measure. In addition to this continuous score, we constructed a quartile variable to examine graded patterns.

#### 2.2.2. Cumulative Resources Inventory

The cumulative resources inventory included 11 indicators created from the items listed in Table 2. The indicators for resources included measures of: (1) social connections (3 Likert-style items); (2) social support (4 count items, modified from a variety of scales [34,35,36]); (3) resilience (4 Likert-style items); (4) positive religious coping (2 Likert-style items); (5) purpose in life (3 Likert-style items); (6) self-esteem (4 Likert-style items); (7) mastery (4 Likert-style items); (8) optimism (3 Likert-style items); (9) conscientiousness (4 Likert-style items); (10) family functioning (6 Likert-style items); (11) child routines (e.g., regular bedtime, frequent family dinners (2 count items; items developed for this survey); and (12) partner support (3 Likert-style items) [37]. Table A2 presents the correlations between the protective factors, means, standard deviations, ranges, thresholds for dichotomization for use in the composite score (typically, the top quartile for continuous scores), and Cronbach’s alpha (when appropriate). To create the cumulative resources inventory score, we summed across the 12 indicators described above to create a continuous score. From this continuous score, we also created a quartile variable to examine graded patterns.

#### 2.2.3. Combined Cumulative Stressors and Resources

To consider the co-occurrence of cumulative stressors and resources, we constructed a four-category variable to capture combinations of high and low scores on the cumulative stressors and resources inventories. For both inventories, we grouped quartiles 1 and 2 as “low” and quartiles 3 and 4 as “high” and created categories of (1) high stressors/low resources, (2) high stressors/high resources, (3) low stressors/low resources, and (4) low stressors/high resources.

#### 2.2.4. Parental Well-Being Outcomes

The survey included brief measures of the caregiver’s anxiety symptoms, depressive symptoms, and sleep difficulties. All three of these outcomes are important for sensitive and responsive parenting [38,39,40]. Anxiety symptoms were measured using the two screening items from a seven-item measure of generalized anxiety disorder (GAD) symptoms (i.e., the GAD-7, a validated and efficient tool for identifying anxiety disorder and severity) [41]. We used the mean of the two screening items to have scores for all participants (Cronbach’s alpha = 0.86), as only those who screened as eligible for GAD received all seven items. The survey assessed depression using 7 Likert-style items (Cronbach’s alpha = 0.64) from the eight-item version of the Center for Epidemiologic Studies Depression scale [42]. In the present analysis, we omitted one item due to high missingness resulting from an error in our electronic survey instrument (i.e., My sleep was restless). The survey measured sleep difficulties in the past four weeks using a three-item measure, with two items taken from the Alameda County Study and one item from the Women’s Health Stress Study [43]. Respondents rated each of the three items on a four-point scale ranging from rarely/never to almost every day (Cronbach’s alpha = 0.65).

#### 2.2.5. Child Medical Record Diagnoses

We abstracted children’s ICD-9- and ICD-10-based diagnoses from the electronic health record (EHR) and generated four dichotomous outcomes, including: (1) pediatric growth/nutrition (i.e., any diagnosis of preterm birth, low birth weight, underweight, obesity, loss of weight, or feeding problems in newborns); (2) laboratory abnormalities (i.e., any diagnosis of nutritional anemia, vitamin or nutrient deficiencies, or elevated lead levels or poisoning); (3) developmental/mental health (i.e., any diagnosis of communication disorders, chronic mental or developmental conditions, or disorders of learning or social functioning with onset in childhood); (4) asthma-like symptoms (i.e., any diagnosis of asthma or reactive airways). See Table A3 for ICD-9 and ICD-10 codes for the diagnosis of each illness.

### 2.3. Statistical Analyses

First, we generated descriptive statistics to summarize the sociodemographic characteristics of the study participants. To describe the bivariate relationship between the cumulative stressors and resources inventories and caregiver and child outcomes, we calculated mean scores by quartiles of the inventory scores, and for the four-category variable that combined across stressors and resources (using Student’s *t*-tests and chi-square tests, respectively). As described above, we used quartile variables for the inventory scores (rather than continuous scores) to examine whether graded relationships exist and avoid assumptions of a linear relationship. Next, we applied regression models to estimate associations between caregiver symptoms and child health outcomes (dependent variables) and the continuous cumulative stressors and resources inventory quartiles (independent variables of interest), adjusted for socioeconomic and demographic characteristics. We used linear regressions to model associations for the caregiver outcomes (i.e., continuous symptom scores), adjusted for the respondent’s age, sex, race/ethnicity, highest education in the household, and parental nativity. We did not apply adjusted models for the child outcomes given the sparse outcomes. Finally, we examined associations between the cumulative stressors and resources inventories as continuous scores, included individually and then together in models adjusted for the covariates described above. As a sensitivity analysis, we tested for multiplicative interactions between the continuous stressors and resources scores for the caregiver outcomes only.

### 2.4. Missing Data

Across the variables in our analysis, missing data ranged from 0 to 9 percent, except for depressive symptoms, which had missing values for 23 percent of respondents. We performed multiple imputation of missing observations using SAS, retaining all respondents that had enrolled. This strategy reduces the likelihood of non-response bias and best preserves the original sample characteristics. We generated 10 imputed datasets that were used in all analyses.

## 3. Results

### 3.1. Demographic Characteristics

Of 169 caregivers in our analysis, 81 percent were biological mothers, and there was a similar proportion of boys and girls (see Table 3). Overall, 59 percent of the caregivers were between the ages of 26 to 35 years, and 10 percent were between 18 to 25 years of age. Sixty-three percent of the children were between the ages of 0 to 2 years. Seventy-seven percent of the caregiver respondents were born outside the U.S. With respect to racial/ethnic composition, 39.1% identified as Hispanic, 23.7% as non-Hispanic White, 16.6% as non-Hispanic Black, 12.4% as non-Hispanic Asian, and 8.3% identified as “non-Hispanic other” race/ethnicity. Over one-third of the sample (36.3%) reported that the highest educational degree in the household is a high school degree or less, and 34.7% reported a college degree or higher.

### 3.2. Bivariate Associations between Stressors, Resources, and Health Outcomes

The first panel of Table 4 presents bivariate associations between caregiver and child health outcomes and the cumulative stressors score, with cumulative stress categorized in quartiles. Across the caregiver outcomes of depressive symptoms, anxiety symptoms, and sleep problems, there is a graded relationship between the cumulative stressors scores and the mean symptom scores, with the greatest symptom scores among those caregivers in the highest quartile of the cumulative stressors score. In contrast, there is no evidence of a graded relationship between quartiles of the cumulative stress score and child diagnoses.

The second panel of Table 4 presents bivariate associations between caregiver and child health outcomes and the cumulative resources score. There was a clear inverse dose–response pattern between the cumulative resources score and depressive and anxiety symptoms, with the lowest mean symptom scores among those caregivers classified into the highest quartile of resources. An inverse relationship between cumulative resources and sleep was evident, but the lowest symptom score was for respondents in Quartile 3. Children’s developmental/mental health outcomes displayed a bivariate association with the cumulative resources score, with children in the highest quartile for the cumulative resources score being the least likely to have a diagnosis. We did not observe any pattern for the relationships between the other child outcomes and the cumulative resources score.

The third panel of Table 4 displays the bivariate associations for the combined cumulative stressors and resources variable. Individuals in the high stressors/low resources category had the highest symptom scores for all three caregiver outcomes relative to the other categories, whereas individuals in the low stressors/high resources category had the lowest symptom scores for all three caregiver outcomes. The symptom scores were relatively similar for individuals categorized in the high stressors/low resources and low stressors/high resources groups. We observed an association between the combined cumulative stressors and resources variable and children’s developmental/mental health outcomes, with over half of children (51.7%) who had this diagnosis categorized within the “high stressors/low resources” group; however, we did not observe patterns between this combined variable and the other child diagnostic outcomes.

### 3.3. Adjusted Associations between Stressors, Protective Factors, and Health Outcomes

Table 5 presents the results of adjusted models for caregiver symptoms. Relative to individuals in the lowest cumulative stressors quartile (i.e., lowest stressors category), caregivers in the highest quartile displayed elevated depressive (β = 4.06, 95% Confidence Interval (CI): 2.09, 6.01) and anxiety (β = 2.63, 95% CI: 1.88, 3.38) symptoms and sleep problems (β = 2.65, 95% CI: 0.96, 4.34). The cumulative resources inventory also displayed an inverse graded association with depressive and anxiety symptoms in the adjusted model. In the comparison of symptoms scores for Quartile 1 to Quartile 4, individuals in Quartile 4 showed lower depressive (β = −3.93, 95% CI: −5.46, −2.40) and anxiety (β = −1.91, 95% CI: −2.62, −1.20) symptoms and sleep problems (β = −1.96, 95% CI: −3.51, −0.40), of a slightly smaller magnitude to that observed for the comparison between Quartiles 1 and 4 for the stressors inventory. Finally, in the models that consider the combination of cumulative stressors and resources, relative to individuals with low stressors and high resources, caregivers with high stressors/low resources displayed elevated depressive (β = 3.84, 95% CI: 2.45, 5.19) and anxiety symptoms (β = 2.01, 95% CI: 1.41, 2.60) and sleep problems (β = 2.32, 95% CI: 1.05, 3.61).

Finally, in Table 6, we present associations between the continuous stressors and resources inventory scores and caregiver outcomes, with the stressors and resources scores included individually (Model 1), and then together (Model 2), in adjusted models. For models of depressive and anxiety symptoms and sleep problems, the cumulative stressors and resources inventories displayed associations when included individually and together (i.e., a positive association for the cumulative stressors inventory, an inverse association for the cumulative resources score, see Model 1). Furthermore, although the stressor and resource inventories are correlated (*r* = −0.39, *p* < 0.0001), the scores displayed independent associations with depressive and anxiety symptoms when included together (see Model 2), with the magnitudes of associations only slightly attenuated. In the model with sleep problems as the outcome, only the cumulative stressors score displayed a clear association, with greater stressors associated with more sleep problems, when the two scores were included together. In our sensitivity analyses, we did not find evidence for multiplicative interactions between the continuous stressors and resources scores for any of the outcomes in the adjusted models presented in Table 6.

## 4. Discussion

There are four key findings from our study. First, inventories of cumulative stressors and resources displayed strong associations with caregiver depressive symptoms, anxiety symptoms, and sleep problems, with cumulative stressors associated with worse outcomes, and cumulative resources associated with better outcomes. Second, the associations were evident in models that included stressors and resources together, for depressive and anxiety symptoms. Third, caregivers with high stressors and low resources displayed the highest levels of depressive and anxiety symptoms and sleep problems, indicating the value of considering stressors and resources together. Finally, with respect to children’s diagnoses, we did not observe associations between the cumulative stressors or resources indices and the child outcomes, except for developmental/mental health outcomes, which showed some evidence for associations, although they were not robust. Taken together, our results indicate that our measure of cumulative stressors and resources was related to a range of caregiver outcomes, whereas this was not the case for diagnostic conditions in young children.

The observed associations for the caregiver outcomes are consistent with a broad literature documenting cumulative impacts of stressors and resources for adult mental health [44]. The lack of associations between the inventory of stressors and child diagnoses was unexpected based on the prior literature [6,45] and could be due to the low prevalence of diagnostic outcomes given the young age of the sample, or perhaps inconsistent recording within the EHRs. The children in this study were 5 years old or younger and therefore may not yet exhibit clinically relevant symptoms that would be detected or recorded in EHRs. Although we did not observe strong patterns with the child outcomes assessed in this study (via EHR), extensive research shows that parental mental health has implications for child development [38,46,47,48]; therefore, further development of this assessment may be of value.

The current study builds on a variety of studies that have examined individual types of parental or family characteristics in relation to outcomes in young children and their parents (e.g., parental adverse childhood experiences [45], economic strain [49], discrimination [50], and optimism [51]). Our study included comprehensive assessments of both risk and protective factors, and we contribute to a growing research area to briefly yet systematically assess social and contextual factors that are important to health and well-being in children [21] and adults [52].

While interpreting the results from this study, it is important to consider several limitations. First, the cross-sectional design limited our ability to study the temporal ordering between our study variables, and therefore, this study is not designed to make causal inferences. Second, all child health outcomes were derived from EHRs, which could be incomplete and did not allow for the examination of continuous symptom scores, which may be ideal for young children such as those in this study. More subtle emerging developing deviations (e.g., fear, behavioral problems in preschool or at home, inadaptability to routine changes, etc.) may not be captured by most pediatric assessments or diagnoses. Third, the survey information was linked to the child’s EHR, which contains diagnoses since birth, meaning our analyses cannot document temporal ordering for either child or adult health outcomes. Fourth, all measures of stressors and resources relied on relatively brief caregiver self-reports, and negative experiences may be under-reported, or positive experiences or attributes could be inflated. Fifth, given the design for this pilot study, which relied on convenience sampling, the sample is small and susceptible to selection bias. Finally, we conducted multiple tests because we had three caregiver outcomes and four child outcomes; however, given the sample size of this pilot study, we were not able to adjust for multiple comparisons.

Notably, this study also has several strengths, including (1) the unique community health clinic sample primarily comprised of racial/ethnic minorities and immigrants; (2) the translation of the survey into four languages to facilitate inclusion of non-English speaking families; and (3) the inclusion of numerous reliable and valid brief measures to comprise comprehensive inventories of stressors and resources, in line with the accumulation model, which is supported by the child development literature. In future research, it is important to examine the utility of this measure and other measures of cumulative stressors and resources, within longitudinal study designs that include repeated measures of child development. In addition, it will be informative to consider a broader range of outcomes in young children, beyond diagnostic conditions, including sleep [53], potential biomarkers of stressors [54,55], and child wellbeing outcomes [56,57] (e.g., happiness, self-esteem, and prosocial behaviors).

In conclusion, our study has documented clear associations between stressors and resources and parental outcomes, and these results are complemented by other studies showing the importance of parental wellbeing for optimal child development [58]. Future studies are needed to develop concise stressors and resources inventories using larger samples across a range of contexts, and these measures should be evaluated in relation to prospectively assessed measures of child well-being and symptomatology. This line of research holds promise for advancing the understanding of social determinants of health disparities for parents and children and informing interventions to prevent the emergence of health disparities.

## Figures and Tables

**Table 1:** Measures of stressors.

Perceived Stress in the Past 12 Months	
How stressful have the past 12 months been for you overall?	Likert Scale: Not stressful (0) to Extremely stressful (4)
Major life stressor count	
Death of a child of yours You experienced a serious personal attack or assault Witness to a serious physical attack or assault Illness or accident when you nearly died Your spouse or child nearly died from an illness or accident	No (0), Yes (1) responses; summed to create a count score.
Everyday Discrimination Scale	
You are treated with less courtesy or respect than other people. You receive poorer service than other people at restaurants or stores. People act as if they think you are not smart People act as if they are afraid of you You are threatened or harassed.	Likert scale: Never (1) to At least once a week (5) α = 0.78
Housing instability	
Since you were pregnant with (child’s name), how many times have you moved?	Numeric response
Job instability	
Since you were pregnant with this child, how many different jobs have you had?	Numeric response
Work schedule instability	
Do you know what days or times you will be working week-to-week?	No (0), Yes (1)
Job insecurity	
What are the chances that you will lose your main job in the next couple of years?	Likert style: Not at all likely to (1) to Very likely (4)
Work–life balance stress	
My job leaves me feeling too tired/stressed after work to participate in activities with my friends/family	Likert style: Disagree strongly (1) to Agree strongly (4)
Financial insecurity	
In general, how do your family finances work out at the end of the month?	Likert style: Some money left over (1) to Not enough to make ends meet (3)
Negative religious coping	
During and after life’s most stressful events, I tend to: 1. feel God is punishing me for my sins or lack of spirituality. 2. wonder whether God has abandoned me.	Likert style, reversed: A great deal (1) to Not at all (5) α = 0.71
Neighborhood safety	
How safe is it to walk around alone in (child’s name)’s neighborhood after dark? Is it:	Likert style: Completely safe (1) to Extremely dangerous (4)
Family immigration concerns	
Do you have concerns or worries about immigration issues for you or anyone close to you?	Likert style: A lot (1) to Not at all (4)
Stressful events, during the 12 months preconception	
A close family member was very sick and had to go into the hospital; I had to take care of a seriously ill or disabled member of the family; One of (child’s name)’s parents or guardians died; Someone else very close to me died; I separated or got divorced from my husband or partner; I was apart from my partner due to military deployment or extended work-related travel; I argued with my partner more than usual; My partner was unfaithful to me; I had a major disagreement over child support, custody, or visitation; I experienced domestic violence or unwanted sexual contact; I had serious problems with a family member or close friend; My partner or I went to jail; I lived with someone who was mentally ill or suicidal, or severely depressed or abusing drugs or alcohol for more than a couple of weeks; I had major difficulties finding appropriate child care or day care; I did not have a job for 3 months or longer when I wanted to be working; I was robbed or my home burglarized; My partner lost their job; I lost my job; My partner or I had a cut in work hours or pay; Someone else in my household was unemployed and looking for work for longer than 3 months; I had problems paying the rent, mortgage, or other bills; I sometimes went without seeing a doctor because I could not pay the bill; I was homeless or had to sleep outside, in a car, in a shelter, or in somebody else’s house; There were times when I needed day care or babysitting but did not have money to pay it; I needed basic items for my child (e.g., diapers) but did not have money to pay for them; The food I bought didn’t last and I didn’t have money to get more.	No (0), Yes (1) responses; summed to create a count score.
*Stressful events, during pregnancy*	
Same as above.	
*Stressful events, since child was born*	
Same as above.	
*Parent adverse childhood events*	
Looking back before you were 18 years of age: Did you live with anyone who was depressed, mentally ill, or suicidal? Did you live with anyone who was a problem drinker or alcoholic, or who used street drugs or abused prescription medications? Did you live with anyone who served time or was sentenced to serve time in a prison, jail, or other correctional facility? Were your parents separated or divorced? Did your parents or other adults in your home ever slap, hit, kick, punch or beat each other, or your sibling(s)? Did a parent or adult in your home ever hit, beat, kick, or physically hurt you in any way? Do not include spanking. Did a parent or adult in your home ever swear at you, insult you, or put you down? Did anyone at least 5 years older than you or an adult, ever touch you sexually, or try to make you touch them sexually, or force you to have sex?	Yes, No; summed to create a count score.

**Table 2:** Measures of Resources and Protective Factors.

Social Connections	
Talk on the phone, text, or get together with family, friends, or neighbors Attend church or religious services Attend meetings of any other groups, clubs, or organizations	Likert scale: Never/less than once per week (1) to Daily/almost every day (5) α = 0.27
Social relationships	
How many close friends or relatives do you have: who you could tell your deepest thoughts and feelings? who you could turn to when you need help with your children? who you could count on to loan you $200 if you needed it? who would provide you with a place to live if you needed it?	4 categories: 0, 1–2, 3–5, 6 or more. α = 0.85
Resilience	
During and after life’s most stressful events, I tend to: find a way to do what’s necessary to carry on. know I will bounce back. learn important and useful life lessons. practice ways to handle things better next time.	Likert scale: A great deal (1) to Not at All (5) α = 0.80
Positive religious coping	
During and after life’s most stressful events, I tend to: work together with God as partners. look to God for strength, support, and guidance.	Likert scale, reversed: A great deal (1) to Not at all (5) α = 0.95
Purpose in life	
I have trouble finding peace of mind. I have a sense of direction and purpose in life. When I think about it, I’m not so sure that my life adds up to much.	Likert Scale: Strongly agree (1) to Disagree strongly (4) α = 0.56
Self-esteem	
I take a positive attitude toward myself. On the whole, I am satisfied with myself. I certainly feel useless at times. At times I think I am no good at all.	Likert Scale: Strongly agree (1) to Disagree strongly (4) α = 0.71
Mastery	
I can do just about anything I really set my mind to. When I really want to do something, I usually find a way to succeed at it. Whether or not I am able to get what I want is in my own hands. What happens to me in the future mostly depends on me.	Likert Scale: Strongly agree (1) to Disagree strongly (4) α = 0.75
Optimism	
If something can go wrong for me it will. I hardly ever expect things to go my way. I rarely count on good things happening to me.	Likert Scale: Strongly agree (1) to Disagree strongly (4) α = 0.62
Conscientiousness	
Please indicate how well each of the following describes you: Organized Responsible Hardworking Careless	Likert Scale: A lot (1) to Not at all (4) α = 0.47
Family functioning	
In times of crisis, we can turn to each other for support. Individuals are accepted for what they are. We can express feelings to each other. We feel accepted for what we are. We are able to make decisions about how to solve problems. We confide in each other.	Likert Scale: Strongly agree (1) to Strong disagree (4) α = 0.85
Child routines	
In a typical week, how many nights, 0 to 7, does your family eat dinner together? (Count response) Is there a regular time that (child’s name) (and, your other children) goes to bed on week days? (Yes, No)	5 or more dinners together per week and “yes” to regular bedtime. α = 0.87
Partner support	
I can trust the other caregiver to take good care of (child’s name). He/she/respects the schedules and rules I make for (child’s name). I can count on the other caregiver for help when I need someone to look after (child’s name) for a few hours.	Likert style: Never true (1) to Always true (4) (no partner = 0) α = 0.79

**Table 3:** Caregiver and child demographic characteristics (*n* = 169 caregivers and 169 children) ^1^.

	n	% ^1^
Caregiver Characteristics		
Race/Ethnicity		
Non-Hispanic White	40	23.67
Non-Hispanic Black	28	16.57
Hispanic	66	39.05
Non-Hispanic Asian	21	12.43
Non-Hispanic Other	14	8.28
Relationship to child		
Mother	137	81.07
Father or Other	32	18.93
Age		
18–25	17	10.00
26–35	99	58.76
36+	53	31.24
Nativity		
In USA	38	22.60
Outside of USA	131	77.40
Highest Education (household)		
High school/GED or less	61	36.33
Some college/2-year degree	49	29.00
College degree +	59	34.67
Currently employed		
No	72	42.72
Yes	97	57.28
**Child Characteristics**		
Sex		
Male	86	50.89
Female	83	49.11
Age		
0–2 years	106	62.54
>2–6 years	63	37.46

^1^ Values are estimated from 10 imputed data files.

**Table 4:** Bivariate Associations between Caregiver and Child Health Conditions by Stressors and Resources ^1^.

	Cumulative Stressors Inventory				
	Quartile 1 (*n* = 43; 25.44%)	Quartile 2 (*n* = 53; 31.36%)	Quartile 3 (*n* = 44; 26.04%)	Quartile 4 (*n* = 29; 17.16%)	
	Mean (SD)	Mean (SD)	Mean (SD)	Mean (SD)	*p-*Value
Caregiver Symptoms, mean (SD)					
Depressive symptoms	2.46 (0.43)	2.47 (0.45)	3.95 (0.47)	6.69 (0.91)	<0.0001
Anxiety symptoms	0.40 (0.18)	0.82 (0.19)	1.42 (0.28)	2.95 (0.30)	<0.0001
Sleep problems	6.59 (0.52)	6.68 (0.43)	7.60 (0.44)	9.24 (0.60)	0.002
*Child Diagnoses, % (n)*					
Development/Mental health	13.79 (4)	31.03 (9)	27.59 (8)	27.59 (8)	0.249
Pediatric growth/nutrition	26.56 (17)	29.69 (19)	28.12 (18)	15.62 (10)	0.928
Lab abnormalities	22.22 (2)	44.44 (4)	22.22 (2)	11.11 (1)	0.846
Asthma-like symptoms	26.67 (4)	26.67 (4)	33.33 (5)	13.33 (2)	0.896
	**Cumulative Resources Inventory**				
	**Quartile 1** **(*n* = 49; 28.99%)**	**Quartile 2** **(*n* = 50; 29.59%)**	**Quartile 3** **(*n* = 42; 24.85%)**	**Quartile 4** **(*n* = 28; 16.57%)**	
	**Mean (SD)**	**Mean (SD)**	**Mean (SD)**	**Mean (SD)**	***p-*value**
*Caregiver Symptoms, mean (SD)*					
Depressive symptoms	5.11 (0.65)	4.22 (0.50)	2.57 (0.48)	1.23 (0.32)	<0.0001
Anxiety symptoms	2.02 (0.28)	1.44 (0.25)	0.72 (0.22)	0.27 (0.19)	<0.0001
Sleep problems	8.52 (0.49)	7.45 (0.45)	6.36 (0.46)	6.54 (0.60)	0.01
*Child Diagnoses, % (n)*					
Development/Mental health	37.93 (11)	41.38 (12)	17.24 (5)	3.45 (1)	0.070
Pediatric growth/nutrition	28.12 (18)	32.81 (21)	25.00 (16)	14.06 (9)	0.854
Lab abnormalities	33.33 (3)	22.22 (2)	11.11 (1)	33.33 (3)	0.463
Asthma-like symptoms	26.67 (4)	33.33 (5)	20.00 (3)	20.00 (3)	0.944
	**Combined Cumulative Stressors and Resources ^2^**				
	**High Stressors,** **Low Resources** **(*n* = 55; 32.54%)**	**High Stressors, High Resources** **(*n* = 18; 10.65%)**	**Low Stressors,** **Low Resources** **(*n* = 44; 26.04%)**	**Low Stressors, High Resources** **(*n* = 52; 30.77%)**	
	**Mean (SD)**	**Mean (SD)**	**Mean (SD)**	**Mean (SD)**	***p-*** **value**
*Caregiver Symptoms, mean (SD)*					
Depressive symptoms	5.55 (0.60)	3.49 (0.81)	3.56 (0.46)	1.53 (0.31)	<0.0001
Anxiety symptoms	2.33 (0.25)	1.12 (0.46)	0.98 (0.25)	0.34 (0.12)	<0.0001
Sleep problems	8.57 (0.43)	7.28 (0.72)	7.24 (0.52)	6.13 (0.41)	0.002
*Child Diagnoses* ^3^ *, % (n)*					
Development/Mental health	51.72 (15)	3.45 (1)	27.59 (8)	17.24 (5)	0.051
Pediatric growth/nutrition	35.94 (23)	7.81 (5)	25.00 (16)	31.25 (20)	0.755
Lab abnormalities	22.22 (2)	11.11 (1)	33.33 (3)	33.33 (3)	0.913
Asthma-like symptoms	40.00 (6)	6.67 (1)	20.00 (3)	33.33 (5)	0.851

^1^ Results are based on 10 imputed data files. ^2^ The phrase “low stressors” refers to Quartiles 1 and 2 of the Cumulative Stressors Inventory; “high stressors” refers to Quartiles 3 and 4 of the Cumulative Stressors Inventory. “Low resources” refers to Quartiles 1 and 2 of the Cumulative Resources Score; “high resources” refers to Quartiles 3 and 4 of the Cumulative Resources Inventory. ^3^ Child diagnoses refers to whether or not the child was diagnosed with any of the diagnoses in each of the diagnostic categories.

**Table 5:** Adjusted linear regression models to estimate the associations between stressors and resources inventories and caregiver outcomes (n = 169) ^1^.

	Depression Symptoms	Anxiety Symptoms	Sleep Problems
	β (SE)	β (SE)	β (SE)
Cumulative Stressors Inventory			
Quartile 1	Reference	Reference	Reference
Quartile 2	0.08 (0.65)	0.46 (0.27)	0.17 (0.68)
Quartile 3	1.54 (0.66) *	0.99 (0.35) **	0.86 (0.72)
Quartile 4	4.06 (0.96) ***	2.63 (0.39) ***	2.65 (0.85) **
Cumulative Resources Inventory			
Quartile 1	Reference	Reference	Reference
Quartile 2	−0.91 (0.69)	−0.69 (0.36)	−1.04 (0.71)
Quartile 3	−2.27 (0.80) **	−1.38 (0.37) ***	−2.10 (0.74) **
Quartile 4	−3.93 (0.76) ***	−1.91 (0.36) ***	−1.96 (0.79) *
Combined Cumulative Stressors and Resources ^2^			
Low Stressors, High Resources	Reference	Reference	Reference
Low Stressors, Low Resources	1.82 (0.65) **	0.74 (0.30) *	1.12 (0.69)
High Stressors, High Resources	1.88 (0.90) *	0.83 (0.49)	1.24 (0.90)
High Stressors, Low Resources	3.84 (0.67) ***	2.01 (0.30) ***	2.32 (0.65) ***

SE = standard error; * <0.05, ** <0.01, *** <0.0001. ^1^ Results are based on 10 imputed data files; linear regression models adjusted for caregiver’s age, race/ethnicity and nativity, and highest educational level in the household. ^2^ The phrase “low stressors” refers to Quartiles 1 and 2 of the Cumulative Stressors Inventory; “high stressors” refers to Quartiles 3 and 4 of the Cumulative Stressors Inventory. “Low resources” refers to Quartiles 1 and 2 of the Cumulative Resources Score; “high resources” refers to Quartiles 3 and 4 of the Cumulative Resources Inventory.

**Table 6:** Adjusted linear regression models to estimate associations between stressors and resources inventories and caregiver outcomes, included separately and together (n = 169) ^1^.

	Depression Symptoms		Anxiety Symptoms		Sleep Problems	
	Model 1 ^a^	Model 2 ^b^	Model 1 ^a^	Model 2 ^b^	Model 1 ^a^	Model 2 ^b^
	β (SE)	β (SE)	β (SE)	β (SE)	β (SE)	β (SE)
Cumulative Stressors Inventory	0.49 (0.10) ***	0.36 (0.10) **	0.30 (0.04) ***	0.25 (0.05) ***	0.36 (0.09) ***	0.30 (0.10) **
Cumulative Resources Inventory	−0.50 (0.10) ***	−0.36 (0.10) **	−0.24 (0.05) ***	−0.14 (0.05) **	−0.28 (0.09) **	−0.16 (0.10)

SE = standard error; ** <0.01, *** <0.0001. ^1^ Results are based on 10 imputed data files. ^a^ Separate models were used to estimate association between each cumulative score and the caregiver outcome, adjusted for caregiver’s age, race/ethnicity and nativity, and highest educational level in the household. ^b^ The cumulative stressors inventory and cumulative resources inventory were included in a single model, adjusted for caregiver’s age, race/ethnicity and nativity, and highest educational level in the household. Notes: The Cumulative Stressors Inventory and Cumulative Resources Inventory are correlated, *r* = −0.39, *p* < 0.0001. The *p*-values for the multiplicative interaction term (cumulative stressors by cumulative resources) were >0.05 for all outcomes.

## Appendix A

**Table A1:** Correlation and Descriptive Statistics for Stressor Measures, original data (prior to multiple imputation).

Variables	1	2	3	4	5	6	7	8	9	10	11	12	13	14	15	16
1. Perceived stress, past 12 months	1.00	**0.16**	**0.28**	**0.32**	**0.26**	**0.16**	**0.18**	**0.18**	**0.14**	0.07	0.04	0.06	0.15	−0.05	0.08	0.08
2. Major life events	0.16	**1.00**	**0.32**	**0.22**	**0.28**	**0.25**	**0.33**	**0.08**	0.07	0.10	0.05	0.03	0.08	0.17	**0.07**	0.17
3. Everyday discrimination	0.28	**0.32**	**1.00**	**0.19**	**0.11**	0.15	**0.26**	**0.18**	**0.12**	−0.06	0.22	**0.29**	**0.06**	0.14	0.16	**−0.01**
4. Stressful events, since birth of child	0.32	**0.22**	**0.19**	**1.00**	**0.42**	**0.38**	**0.24**	**0.15**	0.26	**0.09**	0.01	−0.02	0.21	**0.04**	0.10	0.05
5. Stressful events, during pregnancy	0.26	**0.28**	**0.11**	0.42	**1.00**	**0.51**	**0.19**	**0.10**	0.19	**0.09**	0.02	0.01	0.21	**−0.07**	0.09	0.06
6. Stressful events, year before pregnancy	0.16	**0.25**	**0.15**	0.38	**0.51**	**1.00**	**0.20**	**0.11**	0.13	0.03	−0.03	0.01	0.09	−0.03	0.08	0.04
7. Adverse childhood experiences	0.18	**0.33**	**0.26**	**0.24**	**0.19**	**0.20**	**1.00**	**0.13**	0.10	−0.12	0.18	**0.15**	0.04	−0.04	0.15	0.20
8. Job instability	**0.18**	**0.08**	0.18	**0.15**	0.10	0.11	0.13	1.00	**0.20**	**−0.22**	**0.07**	0.27	**0.14**	0.04	0.20	**−0.03**
9. Residential instability	0.14	0.07	0.12	0.26	**0.19**	**0.13**	**0.10**	0.20	**1.00**	**−0.01**	0.19	**0.01**	0.09	0.05	−0.01	0.12
10. Job schedule instability	0.07	0.10	−0.06	0.09	0.09	0.03	−0.12	−0.22	**−0.01**	1.00	**−0.39**	**−0.55**	**0.23**	**0.06**	−0.12	0.16
11. Job insecurity	0.04	0.05	0.22	**0.01**	0.02	−0.03	0.18	**0.07**	0.19	**−0.39**	**1.00**	**0.56**	**−0.11**	0.08	0.04	−0.01
12. Work family conflict	0.06	0.03	0.29	**−0.02**	0.01	0.01	0.15	0.27	**0.01**	−0.55	**0.56**	**1.00**	**−0.04**	0.08	0.16	**−0.04**
13. Financial strain	0.15	0.08	0.06	0.21	**0.21**	**0.09**	**0.04**	0.14	0.09	0.23	**−0.11**	−0.04	1.00	**0.08**	0.06	0.05
14. Negative religious coping	−0.05	0.17	**0.14**	0.04	−0.07	−0.03	−0.04	0.04	0.05	0.06	0.08	0.08	0.08	1.00	**0.02**	−0.08
15. Unsafe neighborhood	**0.08**	0.07	0.16	**0.10**	0.09	0.08	**0.15**	0.20	**−0.01**	−0.12	0.04	0.16	**0.06**	0.02	1.00	**0.05**
16. Immigration concerns	0.08	0.17	**−0.01**	0.05	0.06	0.04	0.20	**−0.03**	0.12	0.16	**−0.01**	−0.04	0.05	−0.08	0.05	1.00
Sample size	164	165	165	169	169	169	162	147	158	145	168	167	164	165	168	167
Mean	1.68	0.53	2.82	2.77	1.97	1.12	1.27	1.20	0.95	0.52	0.40	0.77	0.80	1.59	0.60	0.95
SD	1.09	1.03	3.48	3.65	2.29	1.79	1.72	1.00	1.34	0.50	0.68	0.97	0.65	2.35	0.69	1.15
Min	0.00	0.00	0.00	0.00	0.00	0.00	0.00	0.00	0.00	0.00	0.00	0.00	0.00	0.00	0.00	0.00
Max	4.00	5.00	19.00	20.00	9.00	9.00	7.00	5.00	11.00	1.00	3.00	3.00	2.00	8.00	3.00	3.00
Highest Quartile Threshold	2.5	1	5	4	3	2	2	2	1	1	1	2	1	3	1	2
Percentage of Obs in Highest Quartile	25.0%	29.1%	26.1%	25.1%	38.6%	35.7%	29.6%	32.7%	53.8%	51.7%	30.4%	25.7%	67.1%	27.9%	49.4%	32.3%
Number of items	1	5	5	26	26	26	8	1	1	1	1	1	1	2	1	1
Reliability	-	-	0.82	-	-	-	-	-	-	-	-	-	-	0.71	-	-

Bolded associations are significant at *p* < 0.05.

**Table A2:** Correlation and Descriptive Statistics for Resources Measures, original data (prior to multiple imputation).

Variables	1	2	3	4	5	6	7	8	9	10	11	12
1. Frequency of social contact	**1**	−0.0419	**0.179673**	**0.406722**	**0.272452**	**0.277569**	**0.207693**	**0.252595**	0.135476	0.141578	0.132751	0.02049
2. Social support	**0.26**	0.13	**0.17**	−0.04	**0.38**	**0.32**	0.09	**0.40**	0.03	**0.28**	**0.310657**	0.116441
3. Resilience score	**0.18**	−0.08	**1**	**0.28**	**0.49**	**0.51**	**0.40**	**0.29**	**0.26**	**0.31**	**0.24039**	0.028193
4. Positive Religious Coping	**0.41**	−0.09	**0.28**	**1**	**0.19**	**0.26**	0.12	0.08	0.11	0.15	−0.01159	−0.06827
5. Purpose in life	**0.27**	0.02	**0.49**	**0.19**	**1**	**0.65**	**0.32**	**0.49**	**0.42**	**0.29**	**0.181122**	0.065679
6. Self-esteem	**0.28**	−0.03	**0.51**	**0.26**	**0.65**	**1**	**0.33**	**0.40**	**0.31**	**0.34**	**0.205096**	0.090047
7. Mastery scale	**0.21**	−0.10	**0.40**	0.12	**0.32**	**0.33**	**1**	0.08	0.15	**0.27**	0.087198	−0.02339
8. Optimism scale	**0.25**	−0.03	**0.29**	0.08	**0.49**	**0.40**	0.08	**1**	**0.16**	0.15	**0.217487**	0.042628
9. Conscientiousness scale	0.14	−0.01	**0.26**	0.11	**0.42**	**0.31**	0.15	**0.16**	**1**	**0.26**	0.036122	−0.00816
10. Family Assessment Device	0.14	0.11	**0.31**	0.15	**0.29**	**0.34**	**0.27**	0.15	**0.26**	**1**	**0.371593**	0.079003
11. Trust in other caregiver	0.13	0.11	**0.24**	−0.01	**0.18**	**0.21**	0.09	**0.22**	0.04	**0.37**	**1.00**	0.08
12. Child routine indicator	0.02	**0.54**	0.03	−0.07	0.07	0.09	−0.02	0.04	−0.01	0.08	0.08	**1.00**
Sample size	163	169	162	167	162	166	161	165	164	166	157	167
Mean	7.16	0.80	12.96	6.45	6.80	9.44	9.48	5.47	10.22	10.23	8.26	0.53
SD	2.97	0.40	3.12	2.71	1.98	2.54	2.32	2.37	1.62	2.18	1.48	0.50
Min	1.00	0.00	3.00	0.00	1.00	0.00	0.00	0.00	4.00	3.00	0.00	0
Max	14.00	1.00	16.00	8.00	9.00	12.00	12.00	9.00	12.00	12.00	9.00	1.00
Highest Quartile Threshold	9	1	16	8	9	12	12	7	12	12	9	1
Percentage of Obs in Highest Quartile	35.0%	29.3%	33.3%	66.5%	32.1%	30.1%	26.1%	33.3%	25.6%	48.2%	71.3%	53.3%
Number of items	3	4	4	2	3	4	4	3	4	4	3	2
Reliability	0.27	0.85	0.80	0.96	0.56	0.71	0.75	0.62	0.47	0.85	0.79	0.87

Note: Bolded associations are significant at *p* < 0.05.

**Table A3:** ICD-10 and ICD-9 codes used to develop child outcomes from electronic health records.

Outcome	Diagnoses	ICD-10 Codes	ICD-9 Codes
Pediatric Growth/Nutrition	Preterm birth	P07.30, P07.20-P07.26, P07.31-P07.39	
	Codes related to low birth weight	P05.00-P05.19, P0700-P0703, P07.10-P07.18	
	Failure to thrive	R62.51	
	Underweight	R63.6, Z68.51	
	Obesity	Z68.53, Z68.54, E66.xx, E66.0x, E66.x (x indicates need for additional digit that indicates BMI percentile)	
	Loss of weight	R63.4	783.21
	Feeding problems in newborn	P92.1-P92.9	779.31
Lab abnormalities	Nutritional anemia	D50.0, D50.1, D50.8, D50.9, D51.0-D51.3, D51.8, D51.9, D52.0, D52.1, D52.8, D52.9, D53.0-D53.2, D53.8, D53.9	
	Vitamin or nutrient deficiencies	E50.*-E64.*	
	Elevated lead levels or poisoning	T56.0x1, T56.0x1, R78.71	
Development/Mental Health	Development/Mental health	F88, F84, F840, F845, F849, F94, F941, F942, F948, F949, F80, F800, F801, F802, F808, F8081, F8082, F8089, F809, F989, F99, F41, F410, F411, F413, F418, F419, F43, F430, F4310, F4311, F4312, F4320-F4325, F4329, F438, F439, F32, F320-F325, F328-F331, F333, F334, F3340, F3341, F3342, F338, F339	
	Communication disorders	F84 Pervasive developmental disorders	31531, 31539; icd10: F801, F809
	The following categories from the chronic condition warehouse if not already included: ADHD, Anxiety, Autism, Intellectual Disability, Learning disability, Developmental Disability, PTSD	F94 Disorders of social functioning with onset specific to childhood and adolescence	
Asthma-like symptoms	Asthma	J4520, J4521, J4522, J4530-J4532, J4540-J4542, J4550-J4552, J45901-J45902, J45909	
	Reactive airways	J68.3	

ICD = International Statistical Classification of Diseases and Related Health Problems; * indicates inclusion of subcategories within the category of diagnosis.
